# Understanding preferences regarding protein-enriched plant-based products of patients with lived experience of (risk of) malnutrition – a grounded theory study

**DOI:** 10.1186/s40795-026-01356-7

**Published:** 2026-05-14

**Authors:** Nathalie Gorter, Harriët Jager-Wittenaar, Marinka Lautenbach, Petra Mulder, Lizette Oudhuis, Annemieke Roelfsema, Martine Sealy

**Affiliations:** 1 Research Group Healthy Ageing, Allied Health Care and Nursing, Hanze University of Applied Sciences, Groningen, The Netherlands; 2 Comprehensive Cancer Center, University Medical Center Groningen (UMCG), Groningen, The Netherlands; 3 Department of Gastroenterology and Hepatology, Dietetics, Radboud university medical center, Nijmegen, The Netherlands; 4 Zorggroep Groningen, Groningen, The Netherlands; 5 Specialized Nutrition, Danone Nutricia Nederland B.V., Rijswijk, The Netherlands; 6 Healthy Food & Nutrition, Hanze University of Applied Sciences, Groningen, The Netherlands; 7 Healthy Food & Nutrition, Van Hall Larenstein University of Applied Sciences, Leeuwarden, The Netherlands; 8 Martini Ziekenhuis, Groningen, The Netherlands

**Keywords:** Malnutrition, Plant-based products, Protein-enriched products, Protein transition

## Abstract

**Background/aim:**

Disease-related malnutrition is common in the Netherlands, and its treatment often involves the use of animal-based protein-enriched products. There is a growing trend and increasing ambition towards a more plant-based diet in the Dutch population and within Dutch healthcare institutions. However, patients may struggle to meet their protein requirements, as the availability of plant-based protein-enriched products remains limited. Patient preferences for such products have not yet been explored. This study aimed to understand patient preferences regarding plant-based protein-enriched products of patients with lived experience of (risk of) malnutrition.

**Methods:**

In this grounded theory study, semi-structured interviews were conducted with patients (≥ 18 years) with recent or current lived experience of (risk of) malnutrition. We systematically analysed the data using open, axial, and selective coding, resulting in themes, using ATLAS.ti and following COREQ guidelines.

**Results:**

Data of 17 out of 18 patients were included in the analysis. Five themes emerged, that addressed product variety, dislikes, health and societal/social considerations, convenience, and packaging. The wish to balance convenience and sustainability gave rise to contradictory product demands. Values of health, sustainability, and animal welfare were similar across participants who were used to eat primarily animal-based and plant-based.

**Conclusion:**

Patients prefer varied, easy-to-eat plant-based products, avoiding strong smells, unhealthy ingredients, and large portions. Emphasizing shared values of health, sustainability, and animal welfare can promote adoption of plant-based options.

**Supplementary Information:**

The online version contains supplementary material available at 10.1186/s40795-026-01356-7.

## Introduction

Disease-related malnutrition is an important health problem and is substantially prevalent across different healthcare settings in the Netherlands (10–35%) [1, 2]. Protein- and energy-enriched products are commonly used in the dietary treatment of malnutrition, as patients with (risk of) malnutrition typically have increased protein and energy requirements [3, 4]. Most protein-enriched products include ingredients derived from animals, such as dairy components, as well as vitamins and minerals sourced from animal raw materials. Although studies show that the development of high-quality protein-enriched plant-based products is now feasible [5, 6] the availability of commercially available plant-based protein-enriched products remains limited. Consequently, patients adhering to predominantly plant-based diets may encounter difficulties in meeting their protein requirements, potentially placing them at greater risk of malnutrition [7].

The global transition towards a more plant-based diet [8], also known as the protein transition, aims to improve human health, while promoting more sustainable use of environmental sources [9–11]. A predominantly plant-based diet can improve public health, by decreasing the risk of several major chronic conditions, such as type 2 diabetes, cardiovascular disease, and cancer [12, 13]. Moreover, the expanding global population increases food demand, putting additional pressure on global resources and the environment. Current food systems are responsible for around a quarter of global greenhouse gas emissions [14], with animal-based proteins having a higher environmental impact than plant-based protein [15]. This underscores the importance of developing plant-based products that meet patients’ protein requirements.

The Dutch Health Council recommends the Dutch population to increase their plant-based protein intake and reduce the consumption of animal-based protein [16]. Currently, 60% of the protein consumed in the Dutch diet comes from animal sources, while 40% comes from plant sources [17]. The Dutch Health Council recommended reversing this ratio to better comply with the national dietary guidelines [16]. Moreover, this transition is expected to reduce the environmental impact of Dutch food consumption by approximately 25%. As a result, many Dutch Healthcare providers aim to achieve a 40:60 ratio of animal-to-plant-based proteins in their menus by 2030 [18].

A survey among Dutch adults showed that 70% of the population supports the protein transition [19] indicating strong motivation to adopt a more plant-based diet. However, the same survey showed that many adults feel unable to start or adhere to such a diet. For patients with (risk of) malnutrition, adhering to a predominantly plant-based diet may be particularly challenging, as nutrition impact symptoms – such as loss of appetite or fatigue - can compromise their ability to adhere to such a diet [20]. Adhering to a prescribed diet can also be challenging, as it requires sufficient intrinsic motivation. Tailoring high-quality, protein-enriched plant-based products to patient preferences may improve acceptance and adherence, thereby supporting the treatment of disease-related malnutrition. However, such preferences have not yet been explored. Therefore, this study aimed to understand the preferences of patients with lived experience of (risk of) malnutrition regarding protein-enriched plant-based products.

## Methods

### Study population

Participants were recruited via Dutch patient associations, newsletters of interest groups, social media platforms and dietitians (Supplement A). The study aimed to explore the preferences of patients with recent or current lived experience of (risk of) malnutrition. Therefore, the inclusion criteria were as follows: adults aged 18 years or older residing at home, in a hospital, or in another healthcare setting; who had an illness at the time of the study or recently before; and who experienced (risk of) malnutrition at the time of the study or recently before. Patients were eligible if they recognised themselves in one or more of the following characteristics indicating being at risk of malnutrition at the time of the study or recently before: unintentional weight loss or looser-fitting clothing; reduction in strength, fitness, and/or muscle mass; eating less than usual; reduced appetite compared to normal; and low weight or a lean appearance. These characteristics were based on the concepts that were used in the development of the Malnutrition Awareness Scale and have been tested as being very comprehensible [21]. Participants’ dietary preferences regarding protein sources, either predominantly animal-based or plant-based, were not an inclusion or exclusion criterion.

### Data collection and ethical approval

Individual, adaptive interviews of 45–60 min were conducted face-to-face, via Microsoft Teams, or by telephone, from January 2024 until July 2024. Interviews were digitally recorded via Microsoft Teams and transcribed verbatim, using a secure transcribing service (ref.nr: 1). All participants signed an informed consent form, approving the recording and transcription of the interviews, as well as the pseudonymised use of the transcripts for scientific publications, prior to participation. Ethical approval was obtained from the University Ethical Advisory Committee, the Hanze Ethische Advies Commissie (heac.T2023.042 A), and the study was conducted in accordance with the principles of the Declaration of Helsinki (2024). The interview guide is shown in Supplement B. The interviews were performed using a semi-structured technique, with each topic having a preconceived main question, followed by a conversation or follow-up question based on the answer. Whenever possible, open questions were asked during the interview. The development of the interview guide was performed by three researchers: a nutritional scientist and clinical dietitian (NG) well experienced with patient contact and malnutrition, and moderately experienced with conducting interviews for qualitative research; a senior researcher specialised in malnutrition and dietetics (MS) and having expertise on the protein transition, and trained and experienced in qualitative research techniques, including conducting, coding and analysing interviews for qualitative research; and a full professor specialised in malnutrition and dietetics (HJW), well experienced in patient care and experienced in qualitative research techniques, including conducting, coding and analysing interviews for qualitative research.

NG performed all interviews. Before conducting the interviews, NG was trained to adopt an open and non-judgmental attitude during consultations with patients. Personal views on plant-based nutrition were consciously set aside during the interviews to maintain neutrality. A practice interview was conducted with an individual who met the participant inclusion criteria. NG and MS extensively evaluated the transcription of this interview, after which the interview guide was adjusted. The quality of the interviews was further assured by asking all participants for feedback on the questions and the interview quality. Since no further points for improvement emerged from the participants’ feedback, no adjustments were made to the interview guide or the interviewing method. Data collection ended after reaching theoretical saturation, i.e., when no new information was provided by the participants that could have led to the development of new selective codes.

### Data analysis

A grounded theory approach, based on Strauss and Corbin’s [22] framework, was employed for data analysis. This process involved three steps: (1) open coding to identify and organise key concepts, (2) axial coding to examine the connections between categories, and (3) selective coding to integrate these categories into a unified theoretical framework [22]. The Consolidated Criteria for Reporting Qualitative Research (COREQ) recommendations for explicit and comprehensive reports of qualitative studies were followed to perform the three stages [23] Firstly, transcripts were imported into the qualitative analysis program ATLAS.ti (Scientific Software Development GmbH, Berlin, Germany). The interviewer and a researcher (MS) co-coded three transcripts, using thematic coding, to create consensus on how the interviews should be open-coded. The interviewer coded the remaining transcripts. Second, the connections between categories were explored by axial coding, which NG and MS collaboratively developed in two sessions. Differences in perspective on coding were discussed to reach consensus on the final axial coding scheme. Third, the perspectives and experiences of the participants were identified and categorised into a thematic framework during selective coding sessions. In five meetings, NG, MS, and HJW together reached consensus on the themes. Quotes were chosen and translated from Dutch to English by NG and MS. HJW proposed a harmonised version of the different translations, which was discussed by NG and MS, after which consensus was reached, and the translation process was finalized.

## Results

Eighteen participants were recruited and interviewed. One participant was excluded because they did not meet any of the characteristics indicating a risk of malnutrition. Data of the remaining 17 participants were included in analysis. Table 1 shows the participants’ characteristics. Of the 17 participants, eight consciously consumed a (predominantly) plant-based diet and nine did not. Eight participants were recruited via the Dutch association for patients with a lung disease (ref.nr: 2).

**Table 1:** Participant characteristics

Total	*N* = 17
Female	13 (76%)
Age category	
20–29 years	3 (18%)
30–39 years	-
40–49 years	1 (6%)
50–59 years	3 (18%)
60–69 years	4 (24%)
70–79 years	5 (29%)
80–89 years	-
90–99 years	1 (6%)
Province	
Groningen	7 (41%)
Noord-holland	4 (24%)
Drenthe	1 (6%)
Flevoland	1 (6%)
Friesland	1 (6%)
Noord-Brabant	1 (6%)
Overijssel	1 (6%)
Utrecht	1 (6%)
Level of education	
Vocational education	3 (18%)
Higher professional education	7 (41%)
Academic education	7 (41%)

Open coding resulted in 239 codes (Supplement C). Axial coding resulted in 24 code groups (Supplement D). A framework consisting of five selective codes (themes) emerged from the coding analyses:

1. Preference for a variety in products and sensory properties.
2. Dislike of product properties.
3. Health-related, societal and social considerations influence dietary choices regarding a plant-based diet.
4. Products should be convenient.
5. Preferences for product packaging and appearance are influenced by societal and social factors.

### Preference for a variety in products and sensory properties

Participants expressed preferences for a variety of products, flavours and textures. The preferred products most frequently mentioned were dairy, chocolate, bars, meat substitutes, cookies, nuts, and warm meals. The preferred products of all participants are listed in Supplement E.

The most frequently mentioned flavours were neutral, sweet, salty, savoury and fresh (Table 2). Preferences varied between softer or liquid textures (Table 3) because they were considered easy to ingest, e.g., *“Eating soup is a lot easier. It sounds weird*,* but the less effort I have to put into it*,* the easier I get it down.” (participant 5).* However, participants also preferred a crunchier texture for a better mouth feel, e.g., *“The bite of a fish stick or something similar*,* I think that’s a good bite. Yeah*,* crispy*,* that is really nice on the outside.” (participant 12)*.

**Table 2:** Preferred flavours for a protein-enriched plant-based product among patients: frequencies from qualitative interview data

Flavour	Frequency
Neutral	8
Sweet	8
Salty	7
Fresh	6
Savoury	6
Fruit	5
Spicy	5
Chocolate	4
Intense	4
Vanilla	4
Full	3
Cheese	3
Coffee	2
Strawberry	2
Orange	2
Herby	2
Fatty	2
Sour	2
Banana	1
Smoky	1
Blueberry	1
Caramel sea salt	1
Cold	1
Nuts	1
Red fruit	1
Mild	1
Mocha	1

**Table 3:** Preferred textures for a protein-enriched plant-based product among patients: frequencies from qualitative interview data

Texture	Frequency
Crunchy	7
Fluid	7
Bite	6
Soft	5
Firm	4
Coarse	4
Al dente	3
Chewy	3
Juicy	2
Seeds	2
Creamy	2
Smooth	2
Sparkling	1
Sticky	1
Fatty	1
Fluffy/airy	1
Thick	1
Soft inside and crunchy outside	1

Participants mentioned a need for variation. They want variation in flavours and textures, both between different products and within meals, e.g., *“When you eat noodles or fried rice*,* you start eating with the taste of fried rice and at the end it still exactly has the same taste. And with a meal*,* I think*,* you have to taste that a carrot is tasty*,* that a piece of potato is tasty*,* I want to taste those separate flavours better.” (participant 1)*,* “At a given point*,* something will start to get unpleasant*,* so when I got the chance to choose other flavours*,* I did.” (participant 14).*

### Dislike of product properties

While the smell of a product was not mentioned in terms of preferences, a dislike for smells, specifically a chemical smell (Table 4), was mentioned, e.g., *“I wouldn’t like that again if it’s another one of those really extreme chemical smells*,* that you know it’s …*,* that the smell is kind of added to it*,* or something.” (participant 17).* Smells can reduce participants’ appetite, e.g., *“Then when I have to cook food and I smell it and things like that*,* it already takes away a lot of my appetite.” (participant 6).*

**Table 4:** Disliked smells for a protein-enriched plant-based product among patients: frequencies from qualitative interview data

Smell	Frequency
Chemical	1
Intense	1

Participants disliked certain products or ingredients due to their perceived adverse health effects. For this reason, they disliked salt, added sugar, saturated fats, and processed foods, e.g., *“Yes*,* I try to eat as little hardened fat as possible. And yet*,* as few fast sugars as possible*,* actually slow sugars. That’s also why I always eat those wholegrain breads. Yes*,* I pay attention to things like that*,* yes.” (participant 10).*

Participants said they feel full quickly and therefore dislike big portions or meals and products that make them feel full, e.g., *“It is important that it is not too much*,* again*,* in terms of volume. And it’s also easy if it doesn’t make you feel so full.” (participant 13)*,* “I do not like whipped cream either. I think that has to do with the texture*,* I think that also has to do with the heaviness of it.” (participant 9).*

Most participants had an aversion to meat or its qualities. They mentioned it takes a lot of chewing, and it is not easy to swallow, and they dislike the bloody appearance, e.g., *‘I won’t eat meat that’s bloody*,* I will never eat steak or roast beef or stuff like that. I would never eat that.’ (participant 6).* This was mentioned by both participants who are used to eat meat and by participants who are not used to eat meat.

Tables 4, 5, and 6 show all disliked smells, flavours and textures, respectively. The disliked products of all participants are listed in Supplement F.

**Table 5:** Disliked flavours for a protein-enriched plant-based product among patients: frequencies from qualitative interview data

Flavour	Frequency
Too sweet	10
Dominant	6
Too salty	5
Fatty	5
Tasteless	4
Taste of soy	2
Too sour	2
Chemical	2
Taste of coffee	2
Bitter	1
Sweetener	1
Taste of mango	1
New tastes	1
Spicy	1
Herbal	1

**Table 6:** Disliked textures for a protein-enriched plant-based product among patients: frequencies from qualitative interview data

Texture	Frequency
Lumpy	3
Thin	2
Syrupy	2
Tough	2
Dry	2
Liquid	2
Stringy	1
Creamy	1
Slimy	1
Watery	1

### Health-related, societal and social considerations influence dietary choices regarding a plant-based diet

Most participants valued animal welfare when it came to their product choices, regardless of whether they (wanted to) eat a plant-based diet or not. This mainly resulted in participants choosing to stop eating products with animal-based ingredients, or to switch to products that they believe provide a more animal friendly alternative, e.g., *“Well*,* we don’t get meat from the supermarket anyway*,* so if we do get it*,* we get it from an “ethical butcher” as we call it here. It may cost a little more if an animal has indeed had a better life.” (participant 8).*

Participants also valued the effect products have on the environment. It was frequently mentioned that the environmental impact of animal products or soy was considered in product choices, e.g., " *That’s again the mind saying: it’s better for the world*,* including the climate. Just better plant-based than dairy. But occasionally I do buy soy desserts*,* but then I feel a bit guilty about that*,* because then people say they are cutting down rainforests anyway.” (participant 2).*

A participant mentioned that they did not like to eat meat because they think meat is unhealthy, e.g., *“When I started preparing meat myself*,* melting the butter and making it completely brown and almost black… I am trained as a doctor*,* and I thought: this can never be healthy. So maybe that is what awakened my reluctance (for meat).’ (participant 12).*

However, other participants were convinced that they needed animal products in their diet to maintain health. Dairy products were the primary choice for this goal. The main reason for this was that animal products, compared to plant-based alternatives, were perceived as providing better nutrients. The nutrients that participants referred to were primarily protein (in terms of both quality and quantity), as well as energy and calcium. Moreover, participants believed that plant-based alternatives were less healthy due to the addition of sugars, and animal-based products were easier to consume. *“I started looking into quarks and protein breakers (protein-enriched yoghurt) to get protein that way. And I find that quite difficult*,* because the plant-based alternatives often contain sugar and less protein and not always calcium. So*,* I don’t think that’s a good alternative.” (participant 15).*

Repeatedly, participants did not know for a fact but had a feeling that plant-based alternatives were insufficient in specific nutrients, e.g., *“Secretly*,* I think that plant protein is of lower quality compared to that from the meat industry anyway. I haven’t looked into it that much*,* and I don’t know if it is true what I’m thinking. But I think it is based on habit.” (participant 4)*,

### Products should be convenient

Participants mentioned that they were often unable to put time and energy into preparing and cooking a meal when they were feeling unwell or fatigued. Therefore, they reported to often eat fast food, even though they would rather eat a healthy home cooked meal, e.g., *“To my shame*,* I have to admit I feel reluctant towards cooking*,* especially when I’m not feeling well*,* so that is when the less healthy things come up.” (participant 4).*

There was also a preference for products that are easy to eat and chew, as these require less time and energy to consume. Participants mentioned that mainly soft and liquid products meet that requirement, e.g., *“I find drinking easier. A kind of yoghurt*,* pudding-like thing*,* I also notice I find that easier to eat. It also spoons up easier*,* like in between meals. If I have to sit down for it*,* I find it difficult; I’m more likely to skip it.” (participant 14).*

Although many participants expressed their preference for something that requires as little effort as possible to prepare, many participants would like to enrich and flavour their food themselves, e.g., “*But a quark-like texture or something*,* which gives you the space*,* if it’s neutral for example*,* to add something to it yourself. Then one person can throw in chocolate sprinkles*,* and another can add fruit. And I think that is very interesting*,* because I find it very difficult that these products all very much have a taste of their own. And no matter how you look at it*,* if you eat it every day*,* you just get done with it very quickly*.*” (participant 9)*,* “I also like it when you have a (protein) powder or liquid*,* that doesn’t matter*,* that you can do something with. A product that you can add to something.” (participant 13).*

Participants mentioned that products should be easy to open and dose. Participants found products easier to use when they could see inside the package. Weighing a product is not preferred because it is seen as too much of a hassle, e.g., “*It should be well proportioned*,* so you can use smaller portions too and are not forced to take a very large quantity*.” (*participant 14)*.

Products should be easy to carry in a bag when leaving home and practical to eat on the way by not melting or falling apart, e.g., *“That you can easily take it with you. That it’s packaged by the piece*,* I think. Easy to put in your bag*,* in your gym bag and can eat a bar immediately after physiotherapy. " (participant 13)*,

### Preferences for product packaging and appearance are influenced by societal and social factors

Participants overall felt that a protein-enriched plant-based product for malnutrition should not stand out or remind them of their illness. It should therefore have a neutral or positive appearance and should not need to be added to their usual diet. Some participants preferred a product that looks like their usual products or a product with which they can enrich their usual products, e.g., *“But I think that’s not really where the benefits are to be gained*,* because there are already a lot of things like that (oral nutritional supplements) and that’s always something you have to take extra or something. Whereas I think*,* if you also look at a kind of more emotional part of food*,* it’s very interesting if you can eat ‘normally’*,* but then get extra filling*,* so to speak. (participant 9)*,

Participants thought that the product itself should be sustainable, but they also mentioned a strong desire for the packaging to be as sustainable as possible. Plastic packaging and products that are individually packaged were considered as bad for the environment by the participants, e.g., “*I have to be honest: I have a lot of trouble with the oral nutritional supplements. There are no straws with it now*,* fortunately*,* but I have terrible trouble with it being packaged like that per bottle. Can it really not be done in half litres? One litre is quite a lot*,* because you’ll be drinking the exact same for two*,* three days …*,* but half litres would be a godsend.” (participant 5).*

There was a need for clear information about the product’s purpose, origin, allergens, and sustainability. Participants expressed that they would like to know whether a plant-based alternative of an animal-based product is equivalent or better in terms of nutrient composition, e.g., *“Well*,* it would be nice if it is clearly indicated*,* like guys*,* this is such a product. And yes*,* I would really like to know how much more protein it contains*,* or if it is equivalent*,* so that I can also make my choice.” (participant 4)*,

### Overarching patterns

Even though participants preferred a product that is easy to eat, which is soft or liquid according to their preferences, they also prefer chewing and experiencing a bite or crunch. This could be contradictory. However, a need for variation, also in terms of texture, was often mentioned. A product with both soft and crunchy features, or a line of products with a variety of textures, could be desirable.

There was a contradiction between the preferences of participants regarding convenience/ease and sustainability in terms of product packaging. Participants preferred single-use packaging because it is easier to carry when leaving home. However, they also rejected single-use packaging because it requires more material and is therefore detrimental to the environment.

While no participant expressed a preference for a specific smell, several participants mentioned a dislike for certain odours. It seemed that a negative smell had a greater influence on product choices or food intake than a positive smell did. This makes it more critical for a product not to have a bad smell than to have a good smell.

Both those who were actively attempting to follow a (mostly) plant-based diet and those who weren’t were dispersed over different age groups. However, in the older age categories, participants were less willing to adjust their product choices towards a more plant-based diet if they had not already made such adjustments. A quarter of male participants deliberately followed a (predominantly) plant-based diet, while a little over half of female participants did. Next to this, there were no apparent observed differences between the participants who did and did not consciously follow a (predominantly) plant-based diet.

The preferences and dislikes mentioned by the participants apply to both plant-based and animal-based products. Participants discussed the good and bad qualities of the products they were eating at that time, regardless of whether they were plant-based or animal-based, and what they would like to see differently if a new product were to be developed. For people who prefer a plant-based diet, the product needed to be completely plant-based. The wishes and needs of participants who prefer animal-based products in their diet seemed not significantly different from those of participants who prefer plant-based products in their diet. Participants who eat (predominantly) plant-based and those who do not shared the same values when it comes to animal welfare, sustainability and health. For example, concerns about animal welfare caused some participants to stop eating meat/dairy products but caused others to switch to organic or animal-friendly’ farmed meat/dairy products.

## Discussion

To our knowledge, this is the first study that aimed to understand the preferences of patients with lived experience of (risk of) malnutrition regarding protein-enriched plant-based products. Patients in our study showed a variety of preferences regarding protein-enriched plant-based products. They expressed a clear preference for variety in products, flavours and textures. Preferences were expressed for both soft and crunchy foods. A dislike was expressed for strong or artificial smells, unhealthy ingredients, and large portions, with patients mentioning an aversion to meat. Surprisingly, patients who consume animal-based products, as well as those who prefer plant-based products shared similar values regarding sustainability, health, and ethical concerns. Patients valued animal welfare and sustainability, while also believing that plant-based products are less optimal than animal products, especially dairy, for meeting nutritional requirements. Older patients may be less willing to adjust their product choices towards a more plant-based diet, unless they have already made such adjustments earlier in life. Preferred were easy-to-prepare and easy-to-eat foods. Packaging that was practical and portable for on-the-go consumption was preferred. Both patients who prefer plant-based products and those who eat animal-based products believe that packaging should be discrete yet not highlighting the illness. They also agree that packaging should look natural: sustainability needs to be emphasised in both the product and its packaging. Clear information on ingredients, allergens, and environmental impact is valued by patients. Many patients in this study would like to consume a more plant-based diet. However, they consciously choose to eat animal-based products, mainly dairy, to ensure proper nutrient intake. Patients fear that plant-based alternatives lack sufficient protein quantity and quality, energy, and calcium, and that they contain excessive amounts of sugar, salt, or saturated fats. Similar studies, on the perceived nutritional adequacy of plant-based alternatives, are scarce and vary in results. One study found that Dutch consumers overestimate the health effects and protein content of plant-based meat alternatives compared to meat products [24]. In contrast, an American study found that most people preferred animal-based dairy products over plant-based dairy products, due to their perceived greater protein content [25].

Several studies have examined the nutritional adequacy of (predominantly) plant-based diets. Diets with traditional plant-based foods (e.g., legumes, vegetables) generally meet nutrient requirements and improve diet quality, whereas those with novel plant-based alternatives (e.g., meat substitutes) may lack key nutrients, such as calcium and B12, and be higher in saturated fat and sodium [26, 27]. Therefore, attention for the nutritional adequacy of novel plant-based products is important. In addition, studies in older adults and patients at risk of malnutrition indicate that well-formulated plant-based oral nutritional supplements are well tolerated and can effectively increase energy, protein, and micronutrient intake, improving nutritional status [28–30].

Our finding that patients express a need for variety is supported by previous research. Danish patients at risk of malnutrition preferred a variety of flavours, as well as a variation in textures and consistencies within meals [31]. Similar findings were reported in older Dutch adults with poor appetite, who demonstrate a strong preference for variety, particularly in high-protein foods, as shown in a computer-based forced-choice food preference assessment [32]. Literature on factors influencing adherence to oral nutritional supplements further highlights that offering a variety of flavours and product types, such as liquids, powders, puddings, and bread formats, may support adherence by reducing taste fatigue and boredom [33]. Additionally, it is shown that greater variety is associated with increased consumption [34].

In this study, nutrition impact symptoms such as fatigue and early satiety emerged as key determinants of patients’ preferences and dislikes. The result show that these nutrition impact symptoms ultimately influence the patient’s product choices. These sensory and physical challenges largely determined what products patients tolerated or avoided. This study found that sensory changes, specifically aversions to intense or artificial smells, can influence product preferences, which is commonly observed in patients receiving cancer treatment [35]. Furthermore, it has been reported that altered sensory perception in patients at risk of malnutrition leads to highly individualised preferences for the sensory qualities of food, which play a crucial role in promoting adequate nutritional intake [31]. Given that alterations in smell and taste can significantly reduce food intake, these factors must be carefully considered in both nutritional interventions and the development of new nutritional products [36]. Addressing nutrition impact symptoms may improve dietary intake, adherence, and, ultimately, nutritional status in patients (at risk of) malnutrition.

Regardless of plant- or animal-based food preferences, patients consistently expressed a desire for convenient, portable products, favouring individually packaged portions for use outside the home in our study. However, concerns about the environmental impact of single-use packaging led to a preference for small, single-portion packs for outdoor use and larger, multi-portion packaging (e.g., 1-litre formats) for indoor consumption. Plastic packaging is generally viewed negatively due to its perceived lack of sustainability. This finding is consistent with previous studies, which show that consumers often perceive plastic as the least sustainable packaging material. At the same time, paper and glass are perceived as more environmentally friendly, primarily based on their recyclability and natural appearance [37, 38].

Some patients reported feeling guilty when consuming soy products due to the belief that soy production contributes to rainforest deforestation. Previous research showed that 47% of Dutch people believe soy cultivation for direct human consumption causes rainforest deforestation, while nearly half feel they know [very] little about soy (49%) [39]. In reality, most soy is used to feed livestock, with only a small fraction consumed directly by humans [40]. Direct consumption of soy-based foods is perceived/considered more resource-efficient than consumption via livestock, reducing land and water use and supporting biodiversity conservation [41]. These findings highlight the need for more accurate information on soy sustainability.

### Strengths and limitations

A strength of this study was the open “bottom-up” conversation structure. This conversation structure ensured a relaxed atmosphere, allowing patients to speak freely and minimising judgment or pressure to steer the conversation in a particular direction.

The study also has some limitations. Firstly, individuals interested in the topic of plant-based diets may have been more inclined to participate, resulting in an overrepresentation of patients that have a positive attitude toward a more plant-based diet. Additionally, a large proportion of participants had an academic educational level, potentially limiting transferability of the results regarding attitude to patients with lower educational backgrounds. Consequently, perceptions of plant-based products in this study may be more positive than those of the general Dutch population. Secondly, patients were included that were at risk of malnutrition at the time of the study or recently before. Therefore, we did not use a validated screening tool to confirm (risk of) malnutrition. Inclusion was based on self-reported signs of malnutrition using criteria from the Malnutrition Awareness Scale (MAS). As a result, some participants may no longer have been malnourished at the time of the interview. However, all had recent lived experience with (risk of) malnutrition, often while managing their nutritional status at home, a setting where maintaining adequate intake is frequently even more challenging. The findings may therefore be particularly transferable to patients with lived experience of (risk of) malnutrition rather than to hospitalised patients with (risk of) malnutrition only. Thirdly, recruitment through an association for patients with a lung disease (ref.nr: 2) may have introduced selection bias, leading to overrepresentation of participants with nutritional impact symptoms related to lung disease. However, reported symptoms such as fatigue and early satiety are common among patients with (risk of) malnutrition in general. Finally, interviews were conducted both online and in-person, which can affect engagement and discussion depth. However, using the same interview guide for both online and in-person interviews ensured that all key topics were consistently discussed, in line with findings from previous research [42].

### Recommendations for research and practice

A key recommendation from this study is that, to facilitate a transition towards increased plant-based protein consumption, it is essential to emphasise the shared moral values—such as concerns for health, sustainability, and animal welfare—rather than focusing on differences in dietary practices or beliefs. Focussing on these common ethical grounds may foster more inclusive and effective strategies for dietary change.

The results of this study suggest that protein-enriched plant-based products should provide a variety of textures and flavours, including both soft and crunchy options, to cater to the sensory preferences of patients.

Products should be designed with consideration for common nutrition impact symptoms, such as fatigue and early satiety, ensuring they are easy to consume, not overly filling, and require minimal preparation. In addition to nutrition impact symptoms, our study identified social considerations that may influence patient’s product choices and preferences. Patients prefer not to be reminded of their illness during meals, and the product should preferably not have an additional character that distinguishes it from ‘normal food’ or ‘regular’ mealtimes. Therefore, it is preferred that a products’ appearance is similar to the appearance of regular snacks or meal components. Products should be convenient for patients at risk of malnutrition and easy to integrate into daily routines. Packaging should avoid plastic, include on-the-go options, and clearly communicate the benefits of the products or ingredients with accurate information on their actual environmental impact.

To promote plant-based protein consumption in patients, novel protein-enriched plant-based products should match or exceed the nutritional quality of the animal-based foods they replace, particularly in terms of protein content. Strong or artificial smells and ingredients perceived as unhealthy (such as saturated fat, salt, and added sugar) should be avoided. Clear labelling of nutritional value and allergens is essential for informed decision-making.

By considering these preferences, patient acceptance of plant-based protein-enriched products may increase. Further research to explore the diverse preferences of different patient groups, based on age, socioeconomic status, and dietary habits, may provide a more detailed understanding of patients’ preferences. This research could include longitudinal studies that introduce new products to patient groups to identify adherence and preferences over time.

## Conclusions

Patients with lived experience of (risk of) malnutrition prefer protein-rich plant-based products that offer a variety of flavours and textures. They dislike strong smells, unhealthy ingredients, and large portions. Regardless of dietary preference, patients value sustainability, health, and ethics, especially animal welfare, but often view plant-based products as less nutritionally adequate than animal-based ones. Convenience and clear labelling on ingredients, allergens, and environmental impact are highly appreciated. Promoting protein-enriched plant-based products among patients may be more effective when emphasizing shared values on health, sustainability and animal welfare.

## Supplementary Information

Supplementary Material 1.

## Data Availability

The pseudonymized interview transcripts generated and analysed during the current study are stored digitally at the Hanze UAS. Due to privacy and confidentiality considerations, these transcripts cannot be made publicly available, as access could compromise participant anonymity. Derived materials, including the quotes and coding framework used in the analysis, are available within the published article (quotes) and its supplementary files (codes).
