# Supplementary material for: Understanding preferences regarding protein-enriched plant-based products of patients with lived experience of (risk of) malnutrition – a grounded theory study
Source: BMC Nutr. 2026 May 14;12:127. doi: 10.1186/s40795-026-01356-7 (PMC13343875; doi:10.1186/s40795-026-01356-7)
Supplement: Supplementary file 1 — Supplementary Material 1. [file 40795_2026_1356_MOESM1_ESM.docx]

# Supplements Understanding preferences regarding protein-enriched plant-based products of patients with lived experience of (risk of) malnutrition

## Supplement A, recruitment

| **Platform** | **Name** | **Way of recruiting** |
| --- | --- | --- |
| Online forum | Veganforum | Recruitment post on the online forum |
| Interest group | Vegetarian Union | Recruitment post on the website |
| Interest group | Dutch Association for Veganism (NVV) | Recruitment post on the website |
| Patient association | SPKS (voor mensen met maag- of slokdarmkanker) | Recruitment post on their website and newsletter. |
| Patient association | Crohn&colitits | Recruitment post on their website and social media. |
| Patient association | Longfonds (Lung Fund) | Recruitment post on their online forum and in their newsletter. |
| Patient association | MDL Fonds | Recruitment post in their newsletter. |
| Facebookgroup | Plantaardige producten NL | Recruitment post in the group. |
| Facebookgroup | Wat eet vegan nederland vandaag | Recruitment post in the group. |
| Facebookgroup | Vegan nederland | Recruitment post in the group. |
| Dietitian | Care group Groningen | Recruitment of patients in care. |
| Dietitian | Martini Hospital Groningen | Recruitment of patients in care. |

##

## Supplement B, interview guide

**Original Dutch version**

| **Topic** | **Doel/aandachtspunten** | **(Mogelijke) vragen** | |
| --- | --- | --- | --- |
| Kennismaking  15 min | Doel: achterhalen welke aspecten maken dat voeding als lekker wordt gewaardeerd  Meenemen in je achterhoofd:  -Uiterlijk (kleur, verpakking, vorm)? Grootte  -Smaak (basis smaken, specifieke smaak, sterkte van de smaak, nasmaak)? Geur (sterkte van de geur)  -Textuur  -temperatuur etc | Wat vindt u nou echt lekker om te eten?  Doorvragen op alle gegeven antwoorden….  Wat vindt u daar nou zo lekker aan?  Smaak: omschrijf wat dit zo lekker maakt?  Bite/structuur: kunt u dit omschrijven?  Verschil in keuzes (bijvoorbeeld bij noten: is er dan nog verschil in noten?  Andere producten: Zijn er nog meer dingen die u lekker vindt om te eten? | |
| Voedingsinname  15 min | Doel: in kaart brengen waar mogelijke bevorderende en belemmerende factoren in de voedingsinname zitten (met betrekking tot ondervoeding en welke aspecten nu juist meer of minder wordt gewaardeerd)    Goed doorvragen op eventuele bevorderende en belemmerende aspecten rondom het eten van producten. | Hoe gaat eten nu? Zijn er dingen die u nu graag eet? Wat maakt dat u dit graag eet? Zit dat in de smaak? …. Textuur … etc  Zijn er ook dingen die u nu minder graag eet?  Wat maakt dat u …. Nu minder vaak eet? Smaak… textuur?  Zijn er ook producten aan u aangeraden om meer van te eten? Waarom? Heeft u dit geprobeerd? Wat vond u? Zijn er ook producten afgeraden? …… | |
| Plantaardige voeding  7 min | Doel: achterhalen hoe de patiënt staat tegenover een (deels) plantaardige voeding.  Welke overwegingen daarbij spelen en of er mogelijk motivatie voor of juist weerstand tegen een (meer) plantaardige voeding aanwezig is. | Indien iemand hiervoor nog **niet** zelf verteld heeft of hij/zij **plantaardig** eet:  In de media wordt regelmatig geadviseerd om meer plantaardig te eten. (bijvoorbeeld door het voedingscentrum). Heeft u hier een mening over? Zo ja, wat vindt u daarvan?  Heeft u wel eens nagedacht om een voeding met meer/overwegend plantaardige producten te gaan eten? (eventueel voorbeelden geven van dierlijke en plantaardige voeding als mensen dit moeilijk vinden)  Zo ja, wat zijn u gedachten daarover? Wat maakt dat u …. Wel of niet belangrijk vindt? | Indien iemand hiervoor **wel** uit zichzelf verteld heeft dat hij/zij (meer) **plantaardig** eet:  U geeft net aan dat u al (voor een deel) plantaardig eet:  In hoeverre eet u bewust plantaardige producten?  Heeft u ook redenen hiervoor? Voorbeelden: duurzaamheid, dierenleed, gemak, kosten, smaak, etc?  Wat vindt u belangrijk aan meer plantaardig eten?  Hoe bevalt het u om (meer) plantaardig te eten? |
| Plantaardige voeding  7 min | Doel: achterhalen in hoeverre de deelnemer klaar is voor de overgang naar een (overwegend) plantaardige voeding --> readiness  Ziekenhuizen gaan over naar een (overwegend) plantaardige voeding, in hoeverre is de patiënt hier klaar voor?  (Er zijn ziekenhuizen die al de overgang maken en wellicht punten hebben waar ze tegen aan lopen). | Als u zich nu eens voorstelt dat u tijdens een periode dat u zorg nodig heeft (meer of alleen maar) plantaardige voeding aangeboden krijgt.  Hoe zou u dat vinden? Heeft u daar ook een mening over?  Wat ziet u daarbij voor u. Hoe stelt u zich een plantaardige voeding voor bij ondervoeding?  Zou u bereid zijn dit te proberen? Indien ja, doorvragen. Indien nee, doorvragen.  Zou u hulp nodig hebben bij een plantaardig dieet? Zo ja, wat voor hulp denkt u nodig te hebben? | |
| Eigenschappen van producten  15 min | Doel: Achterhalen welke vorm, samenstelling, sensorische, milieu, consumptie gerelateerde eigenschappen zou een plantaardig eiwit- en energieverrijkt product moeten hebben?  deelnemer niet sturen en zelf met ideeën laten komen. | Welke dingen vindt u belangrijk aan een plantaardig product bij ondervoeding?  Welke ingrediënten zou u bijvoorbeeld willen (laten) toevoegen? Waarom? Doorvragen.  Houdt iemand van vlees? Wil je dat vervangende producten op vlees lijken? Of juist niet.  Ideeën over gemak, bereiding, samenstelling, hoeveelheid, temperatuur, moment van de dag, houdbaarheid, wat mag het kosten?  Welke ingrediënten zouden lekker zijn? Welke vermijden? Duurzaamheid? Dierenwelzijn?  Verpakking/grootte/hoe vaak? | |
| Afsluiting/samenvatting  1 min |  | Wat vindt u na dit gesprek het meest belangrijk voor ons om mee te nemen in de ontwikkeling van een eiwit- en energieverrijkt product bij ondervoeding?  Heeft u vragen of onderwerpen gemist die graag had willen behandelen in het interview? En wilt u daar nog iets over kwijt?  Heeft u nog vragen?  Wat vond u van het interview?  Heeft u verbeterpunten voor het interview of voor mij? | |

**English translation**

| **Topic** | **Objective / considerations** | **(Possible) Questions / Prompts** | |
| --- | --- | --- | --- |
| Introduction  15 min | Goal: to determine which aspects make food appreciated as tasty  Keep in mind:  - Appearance (color, packaging, shape)? Size  - Taste (basic tastes, specific taste, intensity of the taste, aftertaste)? Smell (intensity of the smell)  - Texture  - Temperature, etc | What do you really enjoy eating?  Follow up on all given answers….  What do you like about that so much?  Taste: describe what makes it so tasty?  Bite/texture: can you describe this?  Difference in choices (for example with nuts: is there still a difference between types of nuts?)  Other products: Are there other things you also enjoy eating? | |
| Food intake  15 min | Objective: to map out where possible promoting and hindering factors in food intake are (with regard to malnutrition and which aspects are currently more or less appreciated)  Ask thoroughly about any promoting and hindering aspects related to eating products. | How is eating going now?  Are there things you currently like to eat? What makes you enjoy eating these?  Is it the taste? ... Texture ... etc.  Are there also things you currently like less?  What makes you eat these less often now? Taste... texture?  Are there also products recommended for you to eat more of? Why? Have you tried this? What did you think?  Are there also products that were advised against? …… | |
| Plant based diet  7 min | Goal: to find out the patient's attitude towards a (partially) plant-based diet. Which considerations are involved? And whether there may be motivation for or resistance to a (more) plant-based diet? | If someone has not yet mentioned whether he/she eats (mostly) plant-based:  The media regularly advises eating more plant-based foods (for example, by the Nutrition Center). Do you have an opinion on this? If so, what do you think about it?  Have you ever considered eating a diet with more / predominantly plant-based products? (possibly give examples of animal and plant-based foods if people find this difficult)  If so, what are your thoughts on this? What makes you find it … important or not? | If someone has **already** mentioned that he/she eats (mostly) plant-based:  You just indicated that you already eat (partly) plant-based:  To what extent do you consciously eat plant-based products?  Do you have any reasons for this? Examples: sustainability, animal welfare, convenience, cost, taste, etc.  What do you consider important about eating more plant-based?  How do you find eating (mostly) plant-based? |
| Plantbased diet  7 min | Goal: determine to what extent the participant is ready for the transition to a (predominantly) plant-based diet --> readiness  Hospitals are transitioning to a (predominantly) plant-based diet, to what extent is the patient ready for this? (Dutch hospitals are already making the transition may have points they are struggling with.) | Imagine if, during a period when you need care, you were offered (more or exclusively) plant-based food. How would you feel about that?  Do you have an opinion on this? What comes to mind? How do you envision plant-based food in the context of malnutrition?  Would you be willing to try this? If yes, ask further. If no, ask further.  Would you need help with a plant-based diet? If so, what kind of help do you think you would need? | |
| Product characteristics  15 min | Goal: Determine what form, composition, sensory, environmental, and consumption-related properties a plant-based protein and energy-enriched product should have.  Do not guide the participants; let the participants come up with ideas themselves. | What do you consider important in a plant-based product for malnutrition?  Which ingredients would you, for example, want to add? Why? Probe further.  (If participant uses animal products:) Do you like meat? Do you want the substitute products to resemble meat? Or not at all.  Ideas about convenience, preparation, composition, quantity, temperature, time of day, shelf life, what should it cost?  Which ingredients would be tasty? Which should be avoided? Sustainability? Animal welfare?  Packaging/size/how often? | |
| Wrap up/summary  1 min |  | After this conversation, what do you think is most important for us to take into account in the development of a protein- and energy-enriched product for malnutrition?  Are there any questions or topics you feel were missed that you would have liked to discuss in the interview? And would you like to add anything about that?  Do you have any questions?  What did you think of the interview?  Do you have any suggestions for improving the interview or for me? | |

## Supplement C, Open codes

## Supplement D, Axial codes

Original Dutch coding

| **Thema 1** | **Thema 2** | **Thema 3** | **Thema 4** | **Thema 5** |
| --- | --- | --- | --- | --- |
| Geur | Geur | Diervriendelijk | Gemakkelijk verkrijgbaar | Natuurlijk/ normaal uiterlijk |
| Product | Gezond | Betaalbaar | Gemakkelijk te eten | Duurzaam |
| Smaak | Voedings-gerelateerde klachten | Gezond | Gemakkelijk in gebruik | Makkelijk mee te nemen |
| Structuur | Product | Milieuvriendelijk | Houdbaar | duidelijke informatie |
| Variatie | Smaak | Sociaal |  |  |
|  | Structuur |  |  |  |

Translated English coding

| **Theme 1** | **Theme 2** | **Theme 3** | **Theme 4** | **Theme 5** |
| --- | --- | --- | --- | --- |
| Smell | Smell | Animal-friendly | Easily available | Natural appearance/normal appearance |
| Product | Healthy | Affordable | Easy to eat | Sustainable |
| Taste | Nutrition-related complaints | Healthy | Easy to use | Easy to take along |
| Structure | Product | Environmentally friendly | Long shelf life | Clear information |
| Variety | Taste | Social |  |  |
|  | Structure |  |  |  |

##

## Supplement E, preferred products

| **Product** | **Frequency** |
| --- | --- |
| Dairy | 7 |
| Chocolate | 6 |
| Bar | 6 |
| Meat substitute | 6 |
| Cookies | 6 |
| Nuts | 6 |
| Warm meal | 6 |
| Fruit | 5 |
| Bread | 5 |
| Vegetables | 5 |
| Cheese | 5 |
| Legumes | 5 |
| Soup | 5 |
| Fish | 5 |
| Meat | 5 |
| Quark | 3 |
| Desserts | 3 |
| Yoghurt | 3 |
| Potato | 2 |
| Crisps | 2 |
| Liquorice | 2 |
| Milk | 2 |
| Pasta | 2 |
| Pure chocolate | 2 |
| Cake | 2 |
| Cereal | 2 |
| Snacks | 2 |
| Soja | 2 |
| Whole wheat bread | 2 |
| Crackers | 2 |
| Snack at coffee time | 2 |
| Asian dishes | 2 |
| Soy yoghurt | 1 |
| Crouton | 1 |
| Chocolate mousse | 1 |
| Fried egg | 1 |
| Oat milk | 1 |
| Ice cream | 1 |
| Plant-based chicken pieces | 1 |
| Buttermilk | 1 |
| Coffee | 1 |
| Mandarin | 1 |
| Pudding | 1 |
| Rice pudding | 1 |
| Tempeh | 1 |
| Hot chocolate | 1 |
| Sausage | 1 |
| Bake-off rolls | 1 |
| Apple syrup | 1 |
| Bonbons | 1 |
| Pie | 1 |
| Bread toppings/spreads | 1 |
| Peas | 1 |
| Fast food | 1 |
| Soft drinks | 1 |
| Liver sausage | 1 |
| Peanuts | 1 |
| Pizza | 1 |
| Salat | 1 |
| Orange | 1 |
| Mashed potato stew | 1 |
| Tea | 1 |
| Tomato | 1 |
| Hot beverages | 1 |
| Water | 1 |
| Sweet bread toppings/spreads | 1 |
| Sugar | 1 |
| Mediterranean dishes | 1 |

## Supplement F, disliked products

| **Product** | **Frequency** |
| --- | --- |
| Meat | 8 |
| Bread | 4 |
| ONS | 3 |
| Meat substitutes | 2 |
| Soda | 1 |
| Cultured meat | 1 |
| Porridge | 1 |
| Raisins | 1 |
| Pork | 1 |
| Tomatoes | 1 |
| Full fat milk | 1 |
| Diner | 1 |
| Dairy | 1 |
| Whole wheat bread | 1 |
| Candy | 1 |
| Soy | 1 |
| Fish | 1 |
| Yoghurt | 1 |
| Hospital food | 1 |
